# Analysis of *Treponema pallidum* subsp. *pallidum* predicted outer membrane proteins (OMPeomes) in 21 clinical samples: variant sequences are predominantly surface-exposed

**DOI:** 10.1128/msphere.00213-25

**Published:** 2025-08-29

**Authors:** Petra Pospíšilová, Pavla Fedrová, Eliška Vrbová, Christopher M. Hennelly, Farhang Aghakhanian, Kelly L. Hawley, Everton B. Bettin, Timothy C. Davenport, Sylvia M. Bruisten, Hélène C. A. Zondag, Philippe A. Grange, Nicolas Dupin, Natasha Arora, Angel A. Noda, Arlene C. Seña, Melissa J. Caimano, Juan C. Salazar, Jonathan J. Juliano, M. Anthony Moody, Justin D. Radolf, Jonathan B. Parr, David Šmajs

**Affiliations:** 1 Masaryk University, Brno, Czech Republic; 2 University of North Carolina (UNC) at Chapel Hill 2331, Chapel Hill, North Carolina, USA; 3 UConn Health 705913, Farmington, Connecticut, USA; 4 Connecticut Children’s, Hartford, Connecticut, USA; 5 Department of Infectious Diseases, Public Health Laboratory, Public Health Service of Amsterdam, Amsterdam, the Netherlands; 6 INSERM, Institut Cochin U1016-CNRS UMR8104, Équipe Biologie Cutané, and GHU AP-HP.Centre, Université de Paris Cité, Hôpital Cochin, Service de Dermatologie-Vénéréologie, CeGIDD, CNR IST Bactériennes, Paris, France; 7 Zurich Institute of Forensic Medicine, University of Zurich 27217, Zürich, Switzerland; 8 Department of Mycology-Bacteriology, Instituto de Medicina Tropical “Pedro Kourí” 115350, Havana, Cuba; 9 Duke University 3065, Durham, North Carolina, USA; The University of Texas Medical Branch at Galveston, Galveston, Texas, USA

**Keywords:** *Treponema pallidum*, syphilis, OMPeome, genetic epidemiology, outer membrane proteins, long-read sequencing, MinION sequencing

## Abstract

The incidence of syphilis, a sexually transmitted disease caused by the *Treponema pallidum* subsp. *pallidum* (TPA), has been surging globally despite effective antibiotic therapy. A new strategy for syphilis control is the development of a multi-component syphilis vaccine with global efficacy, which requires the identification of surface-exposed candidate vaccinogens and the determination of their antigenic diversity within circulating TPA strains. To improve the quality of sequences from repetitive and paralogous regions of the TPA genome, we have developed a sequencing scheme that allows amplification and long-read sequencing of 25 targets encoding TPA proteins including 15 outer membrane proteins. We tested this approach on a set of 21 clinical TPA strains, mostly of European origin preselected by MLST typing. A total of 462 (88%) of 525 amplicons were sequenced. Of 58 new alleles identified in comparison to the SS14 and Nichols TPA reference strains, the majority encoded new protein sequences (*n* = 55; 94.8%). The 55 variant protein sequences were encoded by 99 individual TPA loci, where single amino acid replacements occurred most frequently (*n* = 50), followed by replacements of two to three amino acids (*n* = 35) and differences comprising four or more residues (*n* = 14); the latter included six intra-strain recombination events. Most differences were localized to predicted surface-exposed regions, consistent with adaptive evolution of bacterial determinants that function at the host-pathogen interface. Clinical strains having the same allelic profiles from different localities differed in several loci, suggesting that geographical origin significantly contributes to genetic diversity of circulating strains.

**IMPORTANCE:**

Our findings underscore the importance of analyzing TPA clinical samples isolated from diverse geographical regions in order to understand TPA OMP variability.

## INTRODUCTION

*Treponema pallidum* subsp. *pallidum* (TPA) is the causative bacterial agent of syphilis, a sexually transmitted disease with an incidence of approximately 7 million new cases per year globally (1). Although the introduction of penicillin for the treatment of syphilis in the middle of the last century drastically reduced the number of new cases, during the new millennium, the incidence of syphilis has increased alarmingly in many areas of the world, including the United States, China, and Europe (2). This epidemiologic trend has prompted a search for new control strategies, including the development of a globally efficacious vaccine (3–5). It is generally believed that an effective syphilis vaccine will need to include multiple target antigens. A multi-component vaccine requires not only identification of suitable protein candidates but also determination of their sequence diversity at the protein level in TPA strains currently circulating in human populations.

In recent years, there has been an increasing number of TPA genomes sequenced directly from clinical samples, mostly genital ulcer exudates (6–14). However, in the vast majority of these genomes, the most variable regions are incompletely determined (or missing completely) due to the paralogous and/or repetitive character of the corresponding sequences (15). Although several attempts to determine complete TPA genomes have been made (9, 16–21), such studies have yielded only a small number of complete genomic sequences.

The TPA OMPeome, the syphilis spirochete’s known repertoire of outer membrane proteins (OMPs), includes two OMPs involved in outer membrane biogenesis (BamA/TP0326 and LptD/TP0515), four 8-stranded β-barrel porins (8sβb—TP0126, TP0479, TP0698, and TP0733), five long-chain fatty acid transporters (FadLs—TP0548, TP0856, TP0858, TP0859, and TP0865), four OM factors for efflux pumps (OMFs—TP0966, TP0967, TP0968, and TP0969), and multiple *T. pallidum* repeat (Tpr) proteins (22, 23). In this study, we have developed a target-specific sequencing scheme that allows amplification and long-read sequencing of treponemal determinants encoding for OMPeome components (with the exception of the *tpr* paralogs, the subject of a separate study). Moreover, we have added other targets exhibiting sequence variability in TPA genomes: (i) members of paralogous family 15 comprising fibronectin binding proteins and (ii) non-paralogous proteins TP0304, TP0488, TP0705, and TP0751. Also, two proteins (TP0346 and TP0558) were added based on their ability to discriminate between the Nichols and SS14 clades (24). TP0493 is an invariant locus and was selected as a control. The proposed scheme was applied to 21 clinical strains, mostly of European origin, which had previously been molecularly typed by a multilocus sequence typing (MLST) system, revealing corresponding allelic profiles (APs) (8, 25). Sequencing of OMPeome-encoding chromosomal regions revealed a surprising degree of genetic diversity, even among TPA specimens previously classified as belonging to the same APs.

## MATERIALS AND METHODS

### Samples used in the study

To ensure genetic variability among sequenced genomes, we selected 21 archived samples (from PubMLST database [25]), containing DNAs from TPA strains with predominantly different allelic profiles (Table 1) belonging either to SS14 (*n* = 20) or to Nichols clade (*n* = 1). While the determination of the amount of treponemal DNA copies/μL of samples was not performed due to a very limited volume of clinical samples, the quality and quantity of isolated DNA were assessed based on the performance (positivity rates) of XL-PCR amplifications. Of those, only samples with high amplification success rates (defined as seven positive PCR amplifications out of 10 tested) were used.

**TABLE 1:** Clinical samples and their relevant characteristics

Sample	Clade	Allelic profile^*b*^	Country of origin^*c*^	Year of isolation	MLST database ID (25)
3914/20	SS14	1.1.1	CZ	2020	871
F103	SS14	1.1.1	FR	2016	269
Ams101	SS14	1.1.1	NL	2016	764
L3	SS14	1.1.3	SW	Not available	74
6925/19	SS14	1.1.8	CZ	2019	862
P3	SS14	1.1.8	FR	Not available	NA^*a*^
C66	SS14	1.1.10	Cuba	2014	299
Ams3	SS14	1.1.10	NL	2006	666
3163/16	SS14	1.4.1	CZ	2016	387
F8	SS14	1.11.8	FR	2012	185
F124	SS14	1.17.9	FR	Not available	170
5505/20	SS14	1.26.1	CZ	2020	868
3206/15	SS14	1.28.1	CZ	2015	417
7288/2014	SS14	1.36.1	CZ	2014	429
2822B/18	SS14	1.40.1	CZ	2018	920
Ams25	SS14	1.44.1	NL	2008	699
Ams17	SS14	1.46.3	NL	2007	694
12039	SS14	6.26.1	CZ	2020	8
Ams104	SS14	7.45.9	NL	2016	767
Ams112	SS14	19.3.1	NL	2016	773
Ams90	Nichols	9.7.3	NL	2015	753

^*a*^ “NA” indicates sample not in MLST database.

^*b*^ This AP identification is based on partial gene sequences of three genetic loci, including TP0136, TP0548, and TP0705 (8).

^*c*^ CZ, Czech Republic; FR, France; NL, the Netherlands; SW, Switzerland.

### Amplification of genomic DNA

The DNA of archived samples was amplified using the parallel pooled whole genome amplification protocol as described previously (10). In summary, three parallel reactions using multiple displacement amplification approach (REPLI-g kit, QIAGEN, Valencia, CA, USA) were performed, pooled, and then used as a template for nested PCR amplification of selected regions of interest (Table S1). The DNA was amplified with the PrimeSTAR GXL DNA Polymerase (Takara Bio Inc., Otsu, Japan) using a touchdown PCR with the following cycling conditions: initial denaturation at 94°C for 1 min; eight cycles of 98°C for 10 s, 68°C for 15 s (annealing temperature gradually reduced by 1°C each cycle), and 68°C for 6 min; followed by 35 cycles of 98°C for 10 s, 61°C for 15 s, and 68°C for 6 min (43 cycles in total); and a final extension at 68°C for 7 min. Outer and inner products were amplified using the same protocol. One microliter (µL) of pooled whole genome amplified DNA was used in the first step, and 1 µL of the first-step product was used as template for the second step. Second-step PCR products were subsequently purified using a QIAquick PCR Purification Kit (QIAGEN) and mixed in equimolar amounts for downstream library preparation for Oxford Nanopore sequencing (MinION).

### MinION sequencing of PCR amplicons

Sequencing libraries were prepared using SQK-LSK109 kit (Oxford Nanopore Technologies, Oxford, UK) with barcoding kits EXP-NBD104 and EXP-NBS114, according to the manufacturer’s instructions with the following modifications: incubation on a rotator mixer was done for 10 min (instead of 5 min), and the pellet was resuspended in the elution buffer (EB buffer; QIAGEN) at 37°C (instead of resuspension in nuclease-free water at room temperature). The prepared and pooled libraries were loaded on a Nanopore MinION Spot-on flow cell (FLO-MIN106D, version R9) and sequenced for 48 h. Base calling and de-barcoding steps were done using Guppy (v. 4.4.1) (Oxford Nanopore Technologies) using a high-accuracy base calling model (Q20 was used as a minimal threshold for the read to pass). For each sample, masked reference sequences containing solely tag sequences were prepared using BEDtools (v.2.29.0) (26) and showed only the first and last 200 nucleotides (nts, tags) of regions of interest (ROI)-PCR products. The first and last 200 nt of each MinION read were trimmed using Cutadapt (v1.18) (27), and those sequences were handled as “pair-end reads” (forward and reverse reads). Reads were mapped to masked reference (SS14 and Nichols, respectively) using minimap2 (v2.17) (28). Mapped reads were postprocessed using SAMtools (v.0.1.20) (29), NGSUtils/bamutils (v0.5.9, commit a7f08f5) (30), and Picard (v.2.8.1) (Broad Institute, 2015). Soft-clipped reads with more than 20% of clipped read length and mismatched reads (having more mismatches than 10% of read length) were discarded. Final postprocessing steps were for read length filtering. In the first step, reads with distances between tag sequences smaller than 1,000 nts and greater than 6,000 nts were filtered out (all PCR products including tag sequences fall within range of 1–6 kb). After this step, individual bam files (for each amplicon) were generated according to tag coordinates using SAMtools, and full-length reads were extracted from the MinION basecalled reads using BBMap (v.38.79, sourceforge.net/projects/bbmap/). All subsequent steps were performed for each amplicon (ROI) separately. Amplicon reads were further filtered according to the size of ROI ±500 nt (assuming potential deletion/insertion events) using CutAdapt. Filtered reads were then used for consensus sequence determination.

### Assembly of target sequences and identification of single nucleotide variants (SNVs)

Assembly of target determinants (including OMPeome-encoding determinants) proceeded from consensus generation to consensus polishing steps. Determination of consensus sequence was performed with Canu (v.1.8) (31) using the following parameters: (i) stopping on low coverage (*n* = 1); (ii) random downsampling (*n* = 3,000); and (iii) genome size (*n* = 6 k). If Canu was unable to compute a consensus sequence, the longest sequence from “*”.trimmedReads.fasta.gz file (from trimming step in Canu) was used. Consensus polishing steps were performed using Medaka (0.7.1; https://github.com/nanoporetech/medaka) and Racon (v.1.4.13) (32). Outputs from Canu (either consensus sequence or longest trimmed read) were used as an input for Racon and Medaka and were polished with CutAdapt filtered reads. While Medaka was run with default parameters, Racon parameters were set to the following values: score for matching (*n* = 8), score for mismatch (*n* = −6), gap penalty (*n* = −8), and window length (*n* = 500). Sequences were polished with six approaches, including neural network algorithm Medaka, one to four rounds of Racon algorithm (graph-based algorithm, where consensus of the first round is used as an input for next round of Racon), and Racon-Medaka combination (where output of Racon was used as an input for Medaka as recommended previously by Oxford Nanopore Technologies). SNV identification was verified based on the results of MinION sequencing of 25 targets in the complete genome sequences for reference TPA strains SS14 and Nichols and reference TPE strain Samoa D. Almost 62.5 kbps were analyzed in the set of inner amplicons (*n* = 23) for each of the three tested reference genomes. Out of six different algorithms, the combined analyses of Medaka and Racon-Medaka programs, requiring positive output of both algorithms, resulted in the best sequencing results, with no single nucleotide variant sequence discrepancies in any of the 25 targets and three reference genomes used for verification. On the other hand, a similar approach in the detection of single nucleotide indels was less successful, revealing 142, 139, and 154 falsely identified indels in TPA SS14, TPA Nichols, and TPE Samoa D genomes, respectively. Given this fact, along with our previous experience with MLST of TPA strains (25) in which only two indels were identified in a set of 1,256 sequences of TP0136, 1,321 sequences of TP0548, and 1,436 sequences of TP0705, we omitted identified indels based on the assumption that frameshift mutations in these loci are extremely rare. BUSTED analysis (v4.5) detecting positive selection was performed on the allele alignments using HyPhy v2.5.68 (33, 34).

### Structural mapping of variable OMPeome residues in 3D models

Three-dimensional (3D) models for TP0326, TP0515, and the TPA FadLs (TP0548, TP0858, and TP0865) were generated using AlphaFold3 (35). 3D models for the TPA OMFs (TP0966, TP0967, and TP0968) were obtained from trRosetta, as previously published (22, 36, 37). Amino acid sequences of OMPs from all sequenced strains were aligned using EMBL-EBI Clustal Omega (38). To identify the position of variant residues, we superimposed the alignments onto the corresponding 3D models using UCSF Chimera v1.14 (39), allowing the determination of per-residue conservation values.

## RESULTS

### Amplification and sequencing of TPA selected targets

Twenty-five chromosomal loci (see Table S1 and Materials and Methods) were amplified from TPA DNA samples collected between 2006 and 2020 (Table 1). The samples were taken from 21 syphilis patients in the Czech Republic (CZ; *n* = 8), France (FR; *n* = 4), the Netherlands (NL; *n* = 7), Switzerland (SW; *n* = 1), and Cuba (*n* = 1). Most strains (*n* = 17) represented different APs; however, strains with the same APs were obtained independently from different geographic locations: AP 1.1.1 (*n* = 3) from the Czech Republic, France, and the Netherlands; AP 1.1.8 (*n* = 2) from the Czech Republic and France; and AP 1.1.10 (*n* = 2) from Cuba and the Netherlands. The samples belonged either to SS14 (*n* = 20) or to Nichols clade (*n* = 1).

**Fig 1:**
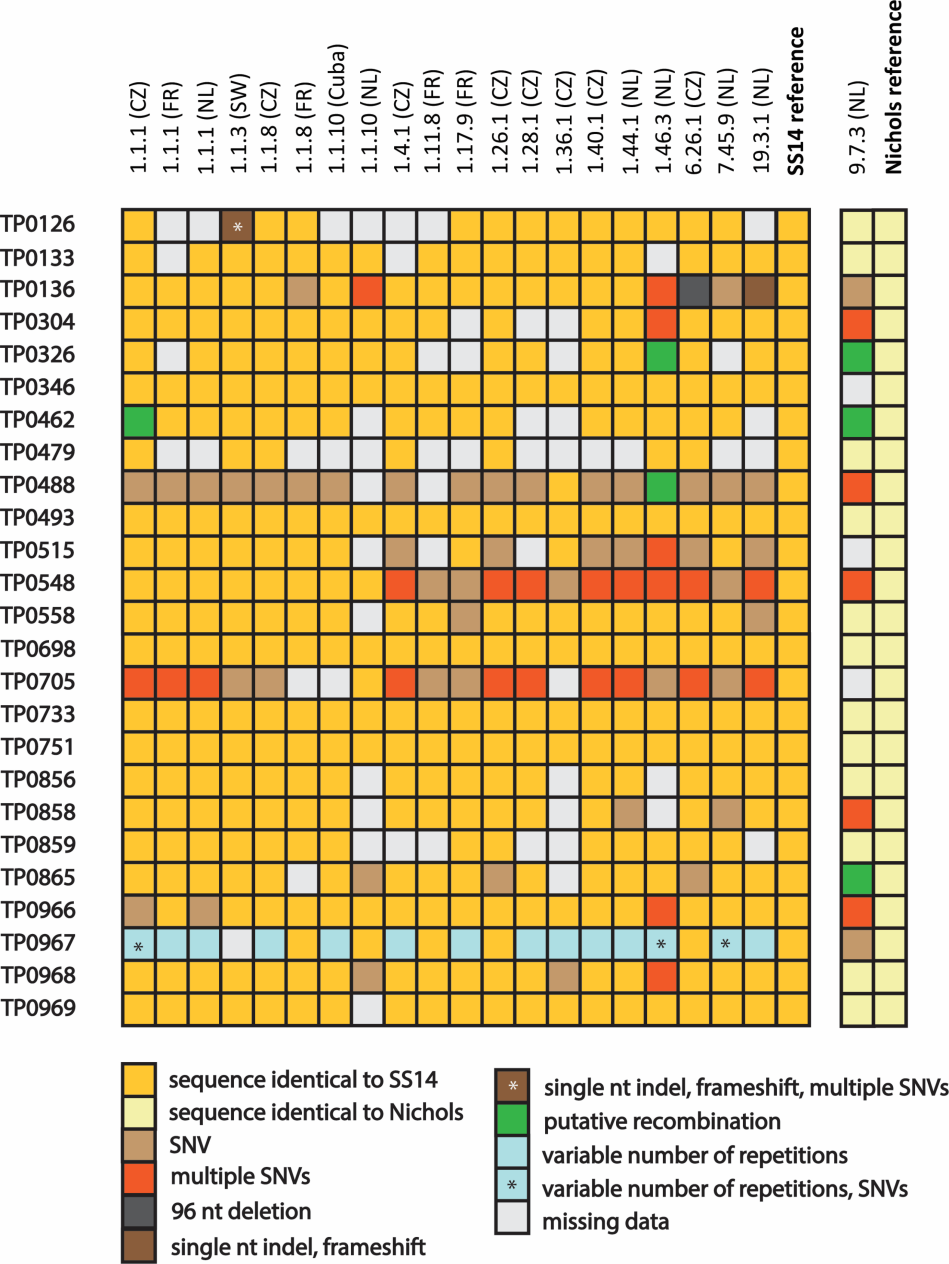
The detected genetic variants in individual loci of 21 TPA specimens. The blank spaces indicate loci (a total of 63 or 12%) that were not analyzed because they either were not amplified or not fully sequenced. Most of the APs (*n* = 20) belonged to the SS14 clade, while one (AP 9.7.3) represented a Nichols-like strain (single column at right). The color code indicates variant loci and type of genetic variant (i.e., presence of SNV, multiple SNVs, 96 nt-long deletion, indel resulting in frameshift mutation, differences in the number of short repetitions, and genetic differences consistent with recombination events).

Of 525 expected amplicons (25 loci analyzed in 21 strains), 462 (88%) were amplified successfully, sequenced, and analyzed, as shown in Fig. 1. All 25 loci were characterized successfully in four strains (APs 1.1.1, 1.1.8, 1.26.1, and 6.26.1; all from the Czech Republic); 23 to 24 loci were characterized in five strains (APs 1.1.1, 1.1.3, 1.40.1, 1.44.1, and 7.45.9 from CZ, SW, and NL); 19 to 22 loci were characterized in ten strains (APs 1.1.1, 1.1.8, 1.1.10, 1.4.1, 1.11.8, 1.17.9, 1.28.1, 1.46.3, 19.3.1, and 9.7.3 from CZ, FR, Cuba, and NL); and 15 to 16 loci were analyzed in two strains (APs 1.1.10 and 1.36.1 from CZ and NL). Amplification efficiencies were lowest for TP0479, TP0126, and TP0859 (analyzed in 8, 14, and 15 specimens, respectively).

### Detected genetic variants within chromosomal loci

Altogether, 58 genetic variants were found among the 462 analyzed loci (shown in Fig. 1). Most of the genetic differences (when compared to the SS14 and Nichols reference strains; Fig. 1) represented single nucleotide variants (SNV; *n* = 49), followed by multiple SNVs (*i.e*., 2-5 SNVs; *n* = 50). In addition, two frameshift mutations (in TP0126 of AP 1.1.3 and in TP0136 of AP 19.3.1), a 96 nt deletion (TP0136 of AP 6.26.1), and 15 differences in the number of short repetitions (nine nt in length in TP0967) were found. Six loci contained larger genetic differences consistent with recombination events (TP0326 in AP 1.46.3, TP0326 in AP 9.7.3, TP0462 in AP 1.1.1*,* TP0462 in AP 9.7.3, TP0488 in AP 1.46.3, and TP0865 in AP 9.7.3). The genetic variability was unequally distributed with no detected variability among 10 loci (TP0133, TP0346, TP0479, TP0493, TP0698, TP0733, TP0751, TP0856, TP0859, and TP0969). These loci included six loci (TP0133, TP0698, TP0733, TP0751, TP0856, and TP0859) that showed no differences between the SS14 and Nichols references.

### Allelic variants at individual chromosomal loci

**Fig 2:**
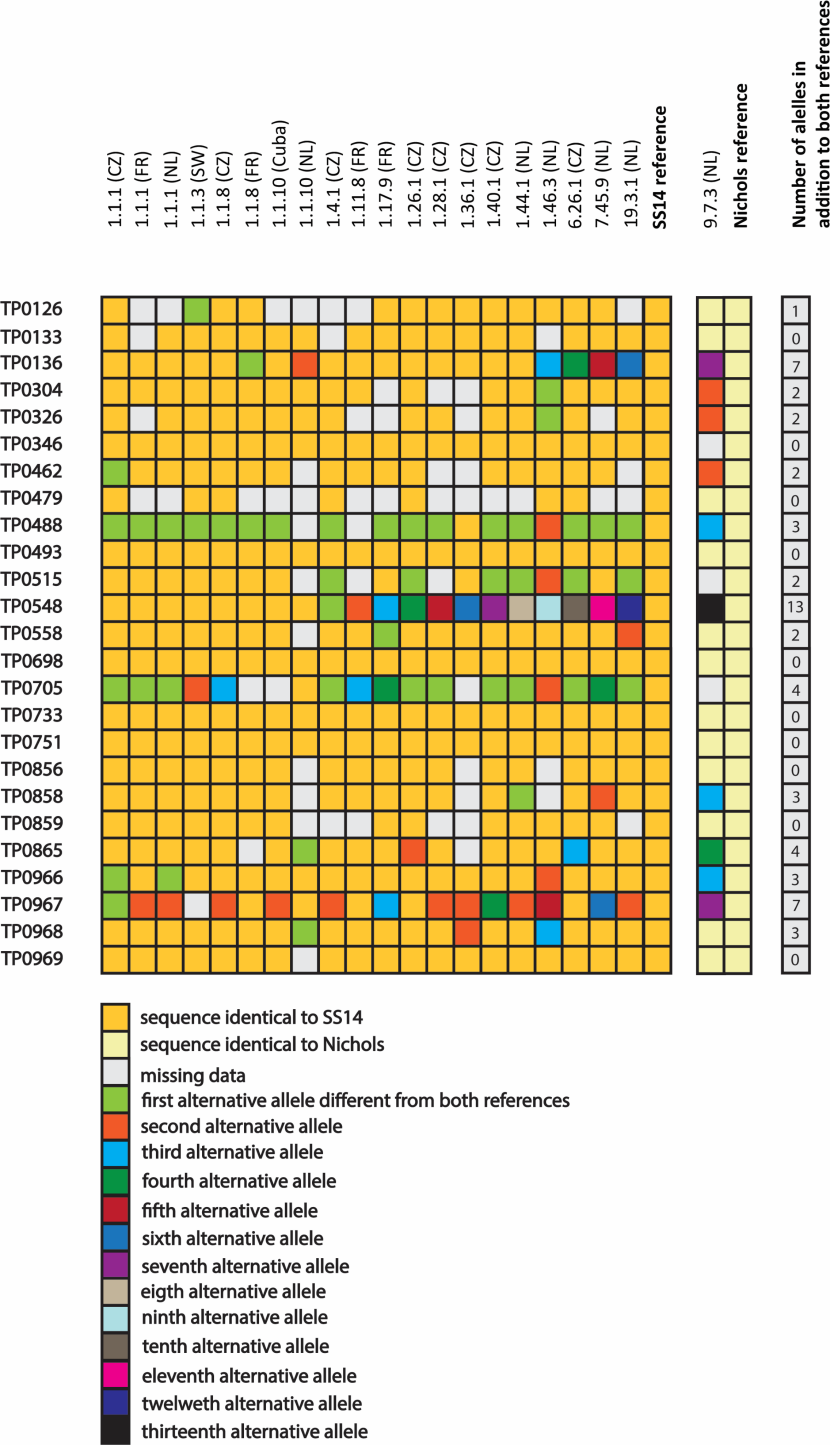
New alleles identified compared to the SS14 and Nichols references, respectively. The number of newly detected alleles in individual profiles ranged between two and 10, with the highest number in AP 1.46.3 (*n* = 10; SS14 clade) and AP 9.7.3 (*n* = 10; Nichols clade). Strains belonging to APs 1.1.1, 1.1.8, and 1.1.10 contained variant alleles.

**Fig 3:**
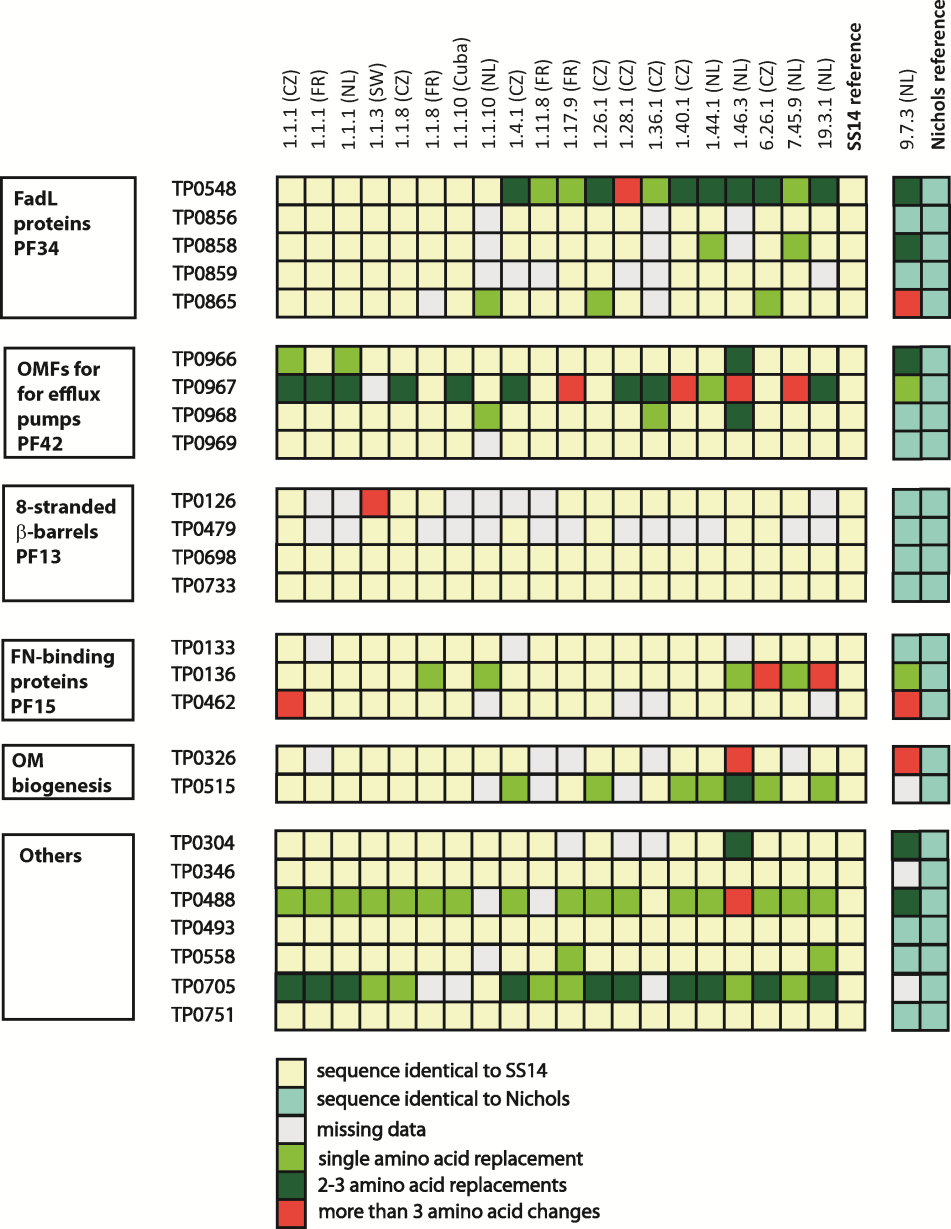
New protein sequences compared to SS14 and Nichols reference genomes. Out of 99 individual loci encoding new protein sequences, single amino acid replacements were most frequent (*n* = 50), followed by replacements of two to three amino acids (*n* = 35) and amino acid differences comprising four or more residues (*n* = 14). The latter group included six intra-strain recombination events and one frameshift mutation (in TP0126). In almost each protein paralogous family, one to two proteins showed remarkably higher diversity compared to other members.

The identified sequences in individual chromosomal loci were compared to the corresponding SS14 or Nichols references. In several loci (*n* = 10), sequences identical to reference sequences were found, including TP0133, TP0346, TP0479, TP0493, TP0698, TP0733, TP0751, TP0856, TP0859, and TP0969, in which there were no alternative alleles. In the remaining 15 loci, the number of alleles with sequences differing from the SS14 and/or Nichols references varied from one (TP0126) to 13 (TP0548) (Fig. 2 and 3). The highest numbers of allelic variants were found for TP0548 (13), TP0136 and TP0967 (7), and TP0705 and TP0865 (4). The number of variant alleles in individual APs ranged between two and 10 with the greatest diversity (10 variants) in AP 9.7.3 (Nichols) and AP 1.46.3 (SS14), both from the Netherlands. Interestingly, strains with the same APs differed at several loci (Fig. 2 and 3). Three strains (AP 1.1.1) from the Czech Republic, France, and the Netherlands differed from each other at three loci (TP0462, TP0966, and TP0967); two strains (AP 1.1.8) from the Czech Republic and France differed at two loci (TP0136 and TP0967); and two strains (AP 1.1.10) from Cuba and the Netherlands differed at four loci (TP0136, TP0865, TP0967, and TP0968).

### Protein variants encoded by new alleles

Nucleotide sequences of individual chromosomal loci were translated to protein sequences and compared to the corresponding SS14 and Nichols references. Of the 58 variant alleles identified, 55 (94.8%) encoded new protein sequences at 99 loci (Fig. 3). The number of new proteoforms in a single TPA strain ranged from four to 12 (median of seven). Two strains from the Netherlands, the SS14-like AP 1.46.3 and the Nichols-like AP 9.7.3, contained the most divergent protein sequences.

Alleles that encoded new protein sequences (*n* = 55) were identified within paralogous protein families (PF) PF34 (FadLs), PF42 (OMFs), and PF15 (TP0136-like lipoproteins) (Fig. 3). Interestingly, in almost each paralogous family, one protein showed remarkably higher sequence diversity compared to other members of the same family. Examples include the FadL TP0548 (13 alleles), the OMF TP0967 (seven alleles), and the fibronectin binding protein TP0136 (seven alleles). Most of the newly detected genetic variants represented transitions resulting in non-synonymous, non-conservative amino acid replacements (Table S2 and S3 in File S1). Four genes (TP0136, TP0488, TP0548, and TP0705) were found positively selected as revealed by BUSTED analysis (Table S2).

### Identification of recombinant loci

Nucleotide sequences of individual chromosomal loci were compared to available treponemal sequences. While five loci were similar or identical to previously identified inter- and intra-strain recombinant variants, one novel recombination event representing a tandem duplication was found in TP0462 of AP 1.1.1 (CZ) (Table 2).

**TABLE 2:** Recombinant loci detected

Gene	AP (country)^*a*^	Similar to corresponding locus of	Sequence identity to previously reported recombinant sequence	Reference
TP0326	1.46.3 (NL)	Mexico A	No differences, identical to Mexico A	(19)
TP0326	9.7.3 (NL)	CW59	1 SNV^*b*^ compared to CW59	(9)
TP0462	1.1.1 (CZ)	Novel intra-gene duplication within TP0462^*c*^	Novel	This work
TP0462	9.7.3 (NL)	CW59	Identical to CW59	(9)
TP0488	1.46.3 (NL)	Mexico A	Identical to Mexico A	(19)
TP0865	9.7.3 (NL)	CW59	6 SNVs compared to CW59	(9)

^*a*^ CZ, Czech Republic; NL, the Netherlands.

^*b*^ SNV, single nucleotide variant.

^*c*^ In 1.1.1 (CZ), a 168-long sequence between coordinates 737 and 904 of TP0462 (coordinates in SS14) is tandemly duplicated (i.e., first repetition between coordinates 737 and 904, second repetition between 905 and 1,072 in TP0462 of 1.1.1 [CZ]). Sequences of both repetitions were found identical to each other and to the SS14 reference sequence.

### Mapping of sequence variants to predicted OMP structures

Identifying the positions of variable residues in OMPs provides valuable insights into the evolutionary pressures at the host-pathogen interface and helps pinpoint conserved, surface-exposed regions that may serve as potential vaccine targets. The TPA OMPeome contains proteins with diverse functions and structures, likely subject to different constraints to their variability (22).

**Fig 4:**
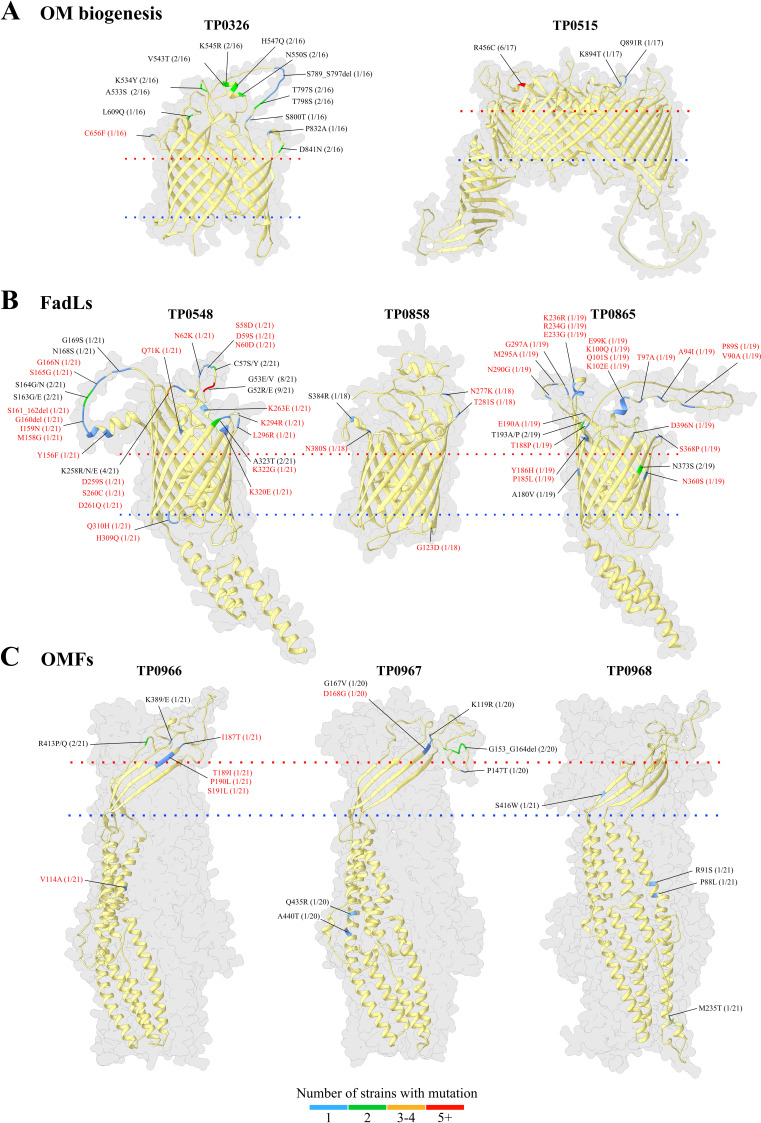
Structural mapping of variable OMP residues identified among sequenced TPA strains. Amino acid sequences of OMPs from all sequenced strains were aligned using Clustal Omega and superimposed onto their corresponding 3D models using UCSF Chimera v1.14. 3D models of TPA orthologs involved in outer membrane biogenesis (**A**) and fatty acid transport-FadL (**B**) were generated using AlphaFold3. Models for outer membrane factors (OMFs) (**C**) were obtained from the trRosetta server. The structure ribbons are color-coded based on the number of sequenced strains containing a variable residue. Differences identified exclusively in the Nichols strain 9.7.3 (NL) are highlighted in red.

We mapped the identified amino acid substitutions onto the predicted AlphaFold3 models of the SS14 reference OMPs (Fig. 4). All variable residues identified in the two OMPs involved in OM biogenesis, BamA/TP0326 and LptD/TP0515 (Fig. 4A), were found in extracellular loops (ECLs). BamA consists of a 16-stranded β-barrel and five periplasmic POTRA domains and is responsible for the insertion of OMPs into the OM of diderm bacteria (40–42). We identified 14 variable amino acid residues distributed in ECLs 3, 4, 5, 7, and 8. Notably, all mutations in TP0326 were found in two strains from the Netherlands: the sole Nichols strain in the study 9.7.3 (NL) and the strain with the previously reported recombination in the TP0326 locus 1.46.3 (NL). TP0515 is an ortholog of LptD, a major component for the assembly of lipopolysaccharides (LPS) in Gram-negative bacteria (43). Curiously, TPA does not contain LPS (44). Moreover, AlphaFold3 predicted a 30-stranded β-barrel for TP0515, larger than prototypical LptD structures (45). A substitution (R456C) localized in ECL5 was more widespread within the analyzed strains (6/17), while two other variable residues in ECL14 (Q891R and K894T) were substitutions found exclusively in a single strain 1.46.3 (NL).

Among the five FadL orthologs for fatty acid transporters, two (TP0856 and TP0859) were fully conserved, while TP0548, TP0858, and TP0865 (Fig. 4B) contained sequence variants when compared to the SS14 reference. Most differences (47/60), however, were exclusively identified in the sole Nichols strain 9.7.3 (NL), reflecting the differences between TPA clades. In FadLs, the N-terminal region of the protein forms a domain that occludes the interior of the barrel and, in TPA orthologs, is predicted to extend into the extracellular milieu (22, 46). In TP0548, prevalent substitutions were found at the N-terminal positions G52 and G53, occurring in nine and eight strains, respectively. Sequence variants in TP0858 occurred in ECLs 4 and 7 and were identified only in individual strains - 1.44.1 (NL), 7.45.9 (NL), or 9.7.3 (NL).

Additionally, we identified 18 variable residues among three of the four OMFs (TP0966, TP0967, and TP0968; Fig. 4C). OMFs are the OM component of Type I secretion system involved in efflux of noxious molecules (45). In addition to their periplasmic helical domains, they feature a 12-stranded β-barrel formed by three identical subunits, each monomer contributing two ECLs. Four and two variable residues were identified, respectively, in ECLs 1 and 2 of TP0966, but only one residue (R413) was variable in more than one strain (2/21). All five surface-exposed mutations in TP0967 were in ECL1, while none of the four substitutions in TP0968 were located in surface-exposed regions.

In total, we identified mutations at 95 different positions across nine of the 15 analyzed OMPs. The vast majority (82/95) of identified mutations were localized to predicted surface-exposed regions supporting the hypothesis that immune pressure drives variability in TPA OMPs.

## DISCUSSION

In this work, we analyzed 25 chromosomal loci in 21 TPA strains that were obtained from samples taken from patients with syphilis. These loci were selected based on proven or potential outer membrane location of the corresponding gene products (22, 23), previous evidence of recombination and adaptive evolution (e.g., TP0488 and TP0326) (47, 48), previous evidence of gene modular structure (e.g., TP0856 and TP0858) (9, 49), binding of extracellular components including fibronectin and laminin (e.g., TP0136 and TP0751) (50, 51), and previous evidence of high SNV density (TP0304, TP0462, and TP0705) (8, 24). Two loci (TP0346 and TP0558) were included based on their ability to differentiate between SS14 and Nichols clades on DNA level (24). Finally, a known invariant locus, TP0493, was selected as a control. To assess genetic and protein variability of clinical TPA strains, we preselected specimens with different TPA allelic profiles or specimens with the same APs but from different countries. The majority of TPA specimens were collected in Europe, including the Czech Republic, France, the Netherlands, and Switzerland, and a single specimen came from Cuba. The analyzed APs thus mostly represent TPA variability with regard to western and middle Europe, known to predominantly contain TPA strains of the SS14 clade (8, 52, 53).

The chromosomal regions analyzed in this work represent highly variable TPA loci often harboring paralogous outer membrane protein gene families. Because of their paralogous and/or highly variable nature, these loci are often completely or partially missing in draft TPA genomes, especially those derived by hybridization capture and Illumina sequencing. In this work, we developed a scheme for target-specific sequencing of such chromosomal regions based on the previously published pooled segment genome sequencing (PSGS) technique (54, 55), wherein individual paralogous regions were amplified with primers recognizing unique chromosomal flanking sequences. Since the length of these regions is often several kbps, we developed a protocol for MinION sequencing where single reads span the entire amplified DNA region to facilitate assembly of these loci. The principal advantages over short-read sequencing technologies include detection of duplications ≥ 150 bp and sequencing of paralogous or recombined regions. The lower quality of MinION sequencing reads (compared to other sequencing platforms) was overcome by selection of algorithms involved in sequence polishing during sequencing of the same genome targets in genomes of strains with known genome sequences (TPA SS14, TPA Nichols, and TPE Samoa D). As a result of this selection process, we used an agreement of two algorithms that gave the best performance in our set of genome targets.

Although all the 25 loci were successfully amplified in four of the 21 strains, several loci appeared to be more difficult to amplify. However, the reasons for poorer amplification of certain loci (TP0479 and TP0136) remain unknown with possible involvement of PCR product length, putative secondary DNA structures within the amplified region, genetic diversity in priming sites, suboptimal primer sequences, and combinations thereof. The high failure rates for the TP0479 locus, one of the largest amplified regions (7,042 nt, Table S1), appear to be due to fragmentation of DNA and/or suboptimal primer binding.

Altogether, 58 genetic variants were identified among 462 analyzed loci of 21 TPA specimens, indicating that approximately every eighth locus has a different genetic variant when compared to the SS14 or Nichols references, an unexpected finding given that TPA is highly clonal (15, 56). However, it is possible that the preselection of clinical strains having different allelic profiles and different geographical origins could have contributed to the observed genetic diversity. Interestingly, strains from different geographical localities with the same allelic profile differed in several loci. This finding suggests that geographical origin may contribute to detected genetic diversity, despite the fact that the distances between sampling sites in European countries are rather small (usually less than 1,000 km) and travel between the sampled countries is unrestricted. On the other hand, each sampled country has a different language(s) and, therefore, might represent a relatively separate epidemiological network. These findings stress the importance of analyzing TPA in clinical specimens from syphilis patients in more remote geographical regions, especially those with limited human travel and possible language separation (14).

In addition to five previously identified recombinant loci that represented inter- and intra-strain recombination events (9, 19, 57), we found one novel intra-gene recombination (i.e., tandem duplication) in TP0462. Since 2012, when the first recombinant sites were described in the TPA Mexico A (19), there has been increasing evidence of this process in treponemal genomes including different *T. pallidum* subspecies (i.e., subsp. *pallidum*, subsp. *pertenue*, and subsp. *endemicum*). To date, over 20 different recombinant treponemal loci (for review, see references 15, 58, 59) encoding treponemal proteins likely involved in host-pathogen interactions were found. While inter-subspecies recombination in *T. pallidum* appears to be relatively uncommon, intra-genomic and intra-genic recombination appears to be more common, one of them shown in this study.

Most interestingly, the vast majority (94.8%) of 58 novel alleles encoded new proteoforms, suggesting that positive selection operates during evolution of these loci. Out of 22 previously identified loci evolving under adaptive evolution, 11 were present in our set of analyzed chromosomal loci (47). Similarly, genes suspected of being positively selected substantially overlapped with our loci (48). In this study, four loci were identified as being under positive selection, including gene TP0705, which was identified for the first time. In many bacterial pathogens, positively selected genes are known to encode proteins involved in the interaction between the host and pathogen, and because of that, the immune pressure ensures that genetic variants are selected to sustain circulation of the pathogen within the host population. Supporting this hypothesis, we demonstrated that the vast majority of sequence variants in OMPs were located in predicted surface-exposed regions. Within each tested protein paralogous family, usually a single protein showed remarkably higher diversity compared to other members of the same family, suggesting that the remaining paralogs may serve as donor sequences for the principal protein of the family and/or that some paralogs are more immunogenic or abundant. Alternatively, the function of the paralogous family is ensured by the stable members providing a degree of redundancy of the variable member of the paralogous family. However, all these possibilities further support the surface localization of the most diverse paralog and therefore its interaction with the host immune system. However, it is not clear if the observed dominance of protein paralog applies also for treponemes of other subspecies, especially in the light of previous findings showing differences in adaptive evolution in genes from different treponemal subspecies (47).

Overall, the findings of our study demonstrate that there is yet undescribed genetic diversity among circulating strains of TPA. Future studies focusing on mapping the temporal and spatial variability of the described loci will be beneficial for the future syphilis vaccine efficiency.

## Data Availability

Raw sequencing reads were submitted to SRA archive under the following accession number Bioproject PRJNA1295804. Determined consensus sequences of 25 loci in 21 samples can be found in Data S1–S25 in File S1.

## SUPPLEMENTAL MATERIAL

File S1 msphere.00213-25-s0001.xlsx Tables S1 through S3 and Data S1 through S25.

ASM does not own the copyrights to Supplemental Material that may be linked to, or accessed through, an article. The authors have granted ASM a non-exclusive, world-wide license to publish the Supplemental Material files. Please contact the corresponding author directly for reuse.
